# Consent mechanisms and default effects in health information exchange in Japan

**DOI:** 10.3389/fdgth.2025.1498072

**Published:** 2025-02-24

**Authors:** Atsushi Ito, Fumihiko Nakamura

**Affiliations:** ^1^ Faculty of Public Policy, Kyoto Prefectural University, Kyoto, Japan; ^2^ Faculty of Engineering, Kitami Institute of Technology, Hokkaido, Japan

**Keywords:** health information exchange, consent mechanism, utility-based consent rate model, default settings, online survey

## Abstract

**Background:**

Health information exchange (HIE) is an information system that efficiently shares patient information across medical institutions. However, traditional consent methods, represented by opt-in and opt-out, face a trade-off between efficiency and ethical, making it difficult to fundamentally improve consent rates. To address this issue, we focused on default settings and proposed an innovative approach called the “two-step consent model,” which leverages the advantages of existing models using utility theory. We evaluated the acceptability of this method.

**Methods:**

An online survey was conducted with 2,000 participants registered with Japan's largest internet survey company. We compared and analyzed the consent rates of the opt-in, opt-out, and two-step consent models.

**Results:**

The opt-in model had a 29.5% consent rate, maximizing patient autonomy but increasing the burden and reducing efficiency. The opt-out model had a 95.0% consent rate but raised concerns among half of the respondents. The two-step consent model had a 68.5% consent rate, demonstrating its cost-effectiveness compared with traditional models.

**Discussion:**

The two-step consent model, involving implicit and explicit consent when needed, ensures efficient consent acquisition while respecting patient autonomy. It is a cost-effective policy option that can overcome the ethical issues associated with the opt-out model. Introducing methods that leverage both opt-in and opt-out advantages is expected to address HIE stagnation.

**Conclusion:**

The two-step consent model is expected to improve consent rates by balancing the efficiency and quality of consent acquisition. To achieve this, patient education is crucial for raising awareness and understanding of HIE and its consent methods.

## 1 Introduction

In recent years, interest in health information exchange (hereinafter HIE) has been growing due to population aging and increased healthcare expenditure worldwide (1). The HIE is an information network that allows medical facilities such as hospitals and clinics to share patient information, including medical records and test results, online. The widespread use of HIE as a medical infrastructure is expected to improve the overall quality and efficiency of medical care, especially by enabling small clinics to provide advanced medical care through enhanced collaboration with large hospitals. Additionally, information sharing among medical facilities could curb duplication of care and overmedication, ultimately conserving medical resources and lessening the economic burden on patients (2, 3). In response to these expectations, the Japanese government has invested a significant amount of R&D funds to establish more than 400 HIEs since the 2000s (4). However, despite the widespread use of these HIEs, the expected results have been slow to materialize. Specifically, the patient registration rate (hereinafter the “consent rate”) and the utilization of services by medical institutions in these HIEs have been stagnant, and the actual number of registered patients (hereinafter the “number of consenters”) is only 1% of the national population (5). Why then do these problems occur? One possible cause is the consent mechanism exchanged between the patients and medical staff. The conventional method of obtaining consent is the opt-in method, in which the default is set to “disagree.” Since the medical staff directly approach patients and explicitly obtain consent through explanation, appeal, and persuasion, a significant burden is placed on both patients and medical staff (6). Thus, these burdens may hinder the improvement of consent rates.

This issue is not limited to Japan but represents a significant policy challenge for countries promoting HIE. In response, the Japanese government has adopted a policy to simplify the consent process through implied consent, such as in-hospital postings, even under the current Personal Information Protection Law. Additionally, it has recommended switching from an opt-in model to an opt-out model to lessen the burden on both patients and medical staff (7). However, in reality, 85.5% of consent acquisition in domestic medical settings is still conducted through direct face-to-face consent forms, indicating that the transition to opt-out has not progressed sufficiently (8). Indeed, the opt-out model, which sets implied consent as the default, automatically considers consent unless the patient explicitly expresses refusal, thereby contributing to the efficiency of consent acquisition and reducing the burden on medical settings.

However, the protection of patient privacy and respect for self-determination (hereinafter the quality of consent acquisition) are not guaranteed. Differences from previous practices in medical settings can cause problems, making the opt-out mechanism not necessarily accepted as a reasonable means (9). This situation suggests a discrepancy between the goal of efficiently registering patient information and the emphasis on the quality of consent acquisition in medical settings, highlighting the necessity of finding a third solution to resolve this dilemma. Indeed, within the domain of medical ethics, there is an extensive body of literature on informed consent, acknowledging significant contributions of previous research in this field. However, prior research in this policy area has focused on the dichotomy between the opt-in and opt-out models, without exploring a third method that leverages the benefits of both (10–12). Conversely, the role of defaults has been extensively examined in behavioral economics. For example, research on nudge theory has demonstrated that default settings significantly influence consumer decision-making and behavior (13). Specifically, studies on status quo bias by Kahneman and Tversky, as part of prospect theory, reveal that people's tendency to adhere to default options stems from a strong psychological inclination to maintain the status quo and resist change (14). However, decisions to join or register for new and unfamiliar services like HIE are not entirely governed by expectations and losses as framed by prospect theory. This perspective does not fully explain why patients express satisfaction with the default option. Therefore, addressing the inefficiencies and ethical challenges of consent acquisition requires reformulating the consent mechanism using utility theory.

Therefore, can the stagnant consent rate be overcome by setting defaults that balance efficiency and quality in obtaining consent? To test this hypothesis, this study mathematically formulates conventional methods of obtaining consent, with a focus on default settings, and introduces a new method of obtaining consent: a two-step consent model that combines opt-out and opt-in mechanisms (hereinafter the “two-step consent model”). Furthermore, we determine its acceptability. Although there are various possible policy options for improving consent rates, policies utilizing default settings are likely to be implemented in society because of their superior cost-effectiveness (15).

The remainder of this paper is structured as follows. Section 2 formulates a model to predict the consent mechanism of patients and the consent rate of the entire population by assuming that each individual acts rationally to maximize his or her utility. We define the model by applying utility theory and summarize the strengths and limitations of the traditional model in terms of balancing efficiency and quality in obtaining consent. In addition, we formulated a model to predict patient consent behavior and the overall consent rate, assuming that individuals act rationally to maximize their utility derived from providing consent for HIE. In Section 3, an online survey is conducted to gauge the acceptability of the two-step consent model under various scenarios, including the traditional model. In this section, we estimate the consent rate for each model based on survey results. In Section 4, we identify the strengths and limitations of the traditional model based on the results of these analyses and evaluate the acceptability of the two-step consent model. Furthermore, we discuss the limitations of this study and highlight areas for future research. Finally, Section 5 summarizes the main conclusions of the study.

## 2 Materials and methods

### 2.1 Assumptions of the consent model

Since the consent rate p is the ratio of the number of persons who have consented to the registration of patient information in the HIE (hereinafter “the number of consenters”) to the number of subjects, it can be obtained through dividing the number of consenters by the number of subjects. For clarity, “subjects” in this context refers to the entire population within the area where HIE is implemented. To improve the sluggish consent rate, it is necessary to understand the consent mechanism. First, the number of consenters on the numerator side is modeled by default, as it is expected to differ depending on the default settings (16). In this context, it is assumed that the decision to register patient information in this HIE is related to the utility of each individual and that the utility depends on the surrounding consent status and the subject's own need for medical care (17), in addition to the time and effort involved in exchanging consent acquisition. The HIE serves as a platform where medical institutions and patients collaborate. As the level of consent increases, so does the value of the subject's own use, leading to a greater willingness to consent and demonstrating a network effect (18). Unlike other platforms, the use of HIE is limited to medical care; therefore, the degree of need for medical care for each individual is an important consideration. However, even if the number of subjects in the denominator is constant, it is debatable whether the scope should be limited to patients or extended to include local residents. In fact, there are various medical information networks, ranging from those used as private goods among specific medical institutions, such as medical malls and healthcare groups, to those regarded as quasi-public goods, mainly in wide areas such as municipalities, medical regions, and entire prefectures (19, 20). If we consider the latter case, it is more reasonable to expand the target audience to include local residents, including patients, because the system is publicly funded and established as a local medical infrastructure. Based on the above, we define the following symbols:

n: The number of subjects, which is assumed to be constant,

S: A set of alternatives. The choices include “agree” and “disagree,”

$s_{i}$: The choice made by each subject *i* from the set of alternatives, ^1^

p: Consent rate (0≤p≤1),

$q_{i}$: Medical necessity of subject (0≤q≤1),

$u_{j}(\;p,q_{i},s_{i})\;(\;j=in,\;out)$: For each model, a utility function that quantifies the utility determined by subject i's choice $s_{i}$ when the consent rate is *p* and subjecti's medical necessity is $q_{i}$ ($0\leq u_{j}\leq 1$),

$U_{j}(p)\;(\;j=in,\;out)$: For each model, the average utility across subjects when the consent rate is *p*.

Utilizing these variables, we construct a model with utility functions to understand the consent mechanisms for each of the previously discussed opt-in and opt-out options.

### 2.2 Definition of the opt-in model

First, we identify the mechanism of the opt-in model. By default, the choice is to “disagree.” Hence, there is no difference in utility for the subjects choosing to “disagree.” That is, for any p,q, we assume $u_{in}(p,q,\mathrm{disagree})=k_{in}$ and $0\leq k_{in}\leq 1$. For agreeing, we assume a monotonically increasing function with respect to p,q. Specifically,

$$\begin{array}{r} u_{in}(p,q,\;\mathrm{agree})\leq u_{in}({\;p}^{\prime },q,\mathrm{agree})\;\;\;\;\;\;\;\mathrm{for}\;\;\mathrm{any}\;q\;\;\;\;\;\mathrm{if}\;\;\;p\leq {\;p}^{\prime },\; \end{array}$$

$$\begin{array}{r} u_{in}(p,q,\;\mathrm{agree})\leq u_{in}(p,q^{\prime },\;\mathrm{agree})\;\;\;\;\;\;\;\;\mathrm{for}\;\;\mathrm{any}\;p\;\;\;\;\;\;\mathrm{if}\;\;\;q\leq q^{\prime }. \end{array}$$

**Figure 1:**
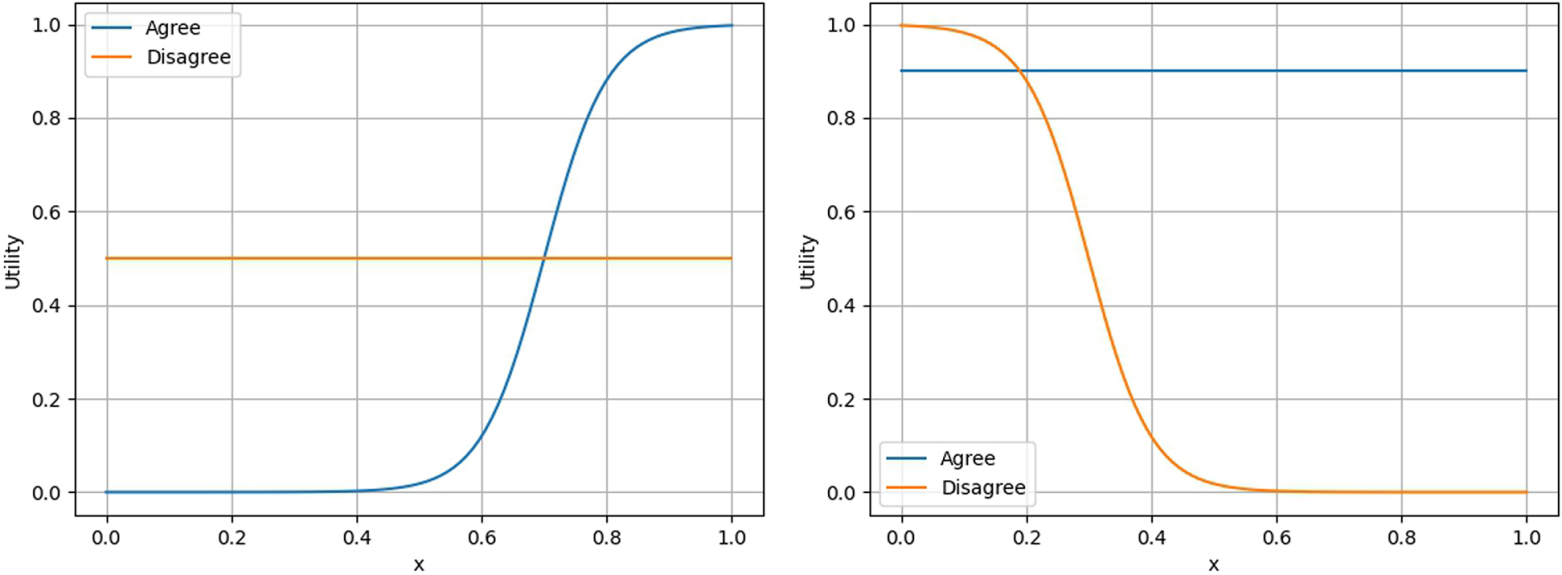
Examples of utility functions in the Opt-in model (left) and the Opt-out model (right). See Supplementary Material for the functions used.

This condition implies that external factors, such as higher consent rates among peers, and internal factors, such as increased personal medical needs, influence the decision to agree. From these assumptions, when p,q are small, $u_{in}(p,q,\;\mathrm{agree})\leq k_{in}$, indicating that the utility of disagreeing is greater, leading to the choice of “disagree.” As p,q increase, there comes a point where $u_{in}(p,q,\;\mathrm{agree})\geq k_{in}$, leading to the choice of “agree” (Figure 1, left side).

This model aims to pre-register patient information in anticipation of future health issues, with medical staff directly approaching patients to explicitly explain the process and obtain consent. Consequently, the quality of consent acquisition is maximized for the patient; however, the process incurs high consent costs for both patients and medical staff due to the need for verbal explanations, appeals, and persuasion, making it inefficient (21). Therefore, the consent rate tends to remain low overall (22).

### 2.3 Definition of the opt-out model

Next, we identify the mechanism of the opt-out model. By default, the choice is to “agree.” Therefore, there is no difference in utility for the subjects choosing to “agree.” That is, for any p,q, we assume $u_{out}(p,q,\;agree)=k_{out}$. For disagreeing, we assume a monotonically decreasing function with respect to p,q. Specifically,

$$\begin{array}{r} u_{out}(p,q,\;\mathrm{disagre})\geq u_{out}({\;p}^{\prime },q,\;\mathrm{disagree})\;\;\;\;\mathrm{for}\;\;\mathrm{any}\;q\;\;\;\mathrm{if}\;\;\;p\leq {\;p}^{\prime }, \end{array}$$

$$\begin{array}{rl} u_{out}(p,q,\;\mathrm{disagree}) & \geq u_{out}(p,q^{\prime },\;\mathrm{disagree})\;\;\;\;\mathrm{for}\;\;\mathrm{any}\;p\;\;\mathrm{if}\;\;\;q\leq q^{\prime }.\; \end{array}$$

This assumption implies that the utility of disagreeing decreases as the surrounding consent rates and personal medical needs increase.

From these assumptions, when p,q are small, $u_{out}(p,q,\;\mathrm{disagree})\geq k_{out}$, that is, the utility of disagreeing is greater, leading to the choice of “disagreeing.” As p,q increase, there comes a point where $u_{out}(p,q,\;\mathrm{disagree})\leq k_{out}$, implying that the utility of agreeing becomes greater, leading to the choice of “agreeing” (Figure 1, right side).

Unlike the opt-in model, this model sets the default to “agree” when registering patient information. Hence, unless the patient explicitly indicates “disagreeing,” consent is assumed automatically. This presumed consent reduces the consent costs compared with the opt-in model, making it more efficient (23). However, it should be noted that this may not always reflect the true intentions of patients and could potentially raise ethical concerns.

### 2.4 Measurement of average utility in the opt-in and opt-out models

Under the above assumptions, the average utility in both models is defined by the following equation:

$$U_{j}(p)=\frac{1}{n}\overset{n}{\underset{i=1}{\sum }}\max\{u_{j}(p,q_{i},s_{i}),\;k_{j}\}\;\;\;\;\;\;(j=in,\;out).$$

This equation calculates the average utility for all subjects i, where i(0≤i≤n) in both models j=in,out, given the surrounding consent rate p and individual medical need $q_{i}$ of subject i when they choose option $s_{i}$. Here, if the utility coefficient is given by

$$u_{j}(p,q_{i},s_{i})=\{\begin{array}{c} 1\;\;(s_{i}=\mathrm{agree})\;\;\;\;\\ \;\;\;0\;\;(s_{i}=\mathrm{disagree})\;, \end{array}$$

then the utility is determined independently of p,q and $U_{j}(p)=p$, indicating that $U_{j}$ matches the consent rate. This represents a simple ratio measurement by counting. Furthermore, $U_{j}(p)$ can be transformed as follows:

$$u_{j}(p,q_{i},s_{i})=\{\begin{array}{c} 1\;\;(s_{i}=\mathrm{agree})\;\;\;\;\\ \;\;\;0\;\;(s_{i}=\mathrm{disagree})\;, \end{array}$$

then the utility is determined independently of p,q and $U_{j}(p)=p$, indicating that $U_{j}$ matches the consent rate. This represents a simple ratio measurement by counting. Furthermore, $U_{j}(p)$ can be transformed as follows:

$$\begin{array}{rl} U_{in}(p) & =\frac{1}{n}\sum_{s_{i}=\mathrm{agree}}\max\{u_{in}(\;p,q_{i},s_{i}),\;k_{in}\}\\ & +\frac{1}{n}\sum_{s_{i}=\mathrm{disagree}}\max\{u_{in}(\;p,q_{i},s_{i}),\;k_{in}\}\\ & =\frac{1}{n}\sum_{s_{i}=\mathrm{agree}}u_{in}(\;p,q_{i},s_{i})+\frac{\#\{s_{i}=\mathrm{disagree}\}}{n}k_{in}\\ & =\frac{1}{n}\sum_{s_{i}=\mathrm{agree}}u_{in}(\;p,q_{i},s_{i})+(1-p)k_{in}. \end{array}$$

Here, we used $u_{in}(p,q_{i},\;\mathrm{disagree})=k_{in}$ and $\#\{s_{i}=\;\mathrm{disagree}\}/n=p.$ Similarly,

$$U_{out}(p)=\frac{1}{n}\sum_{s_{i}=\mathrm{disagree}}u_{out}(p,q_{i},s_{i})+pk_{out}$$

### 2.5 Differences in average utility between the opt-in and opt-out models

Next, we examine the extent of the differences in average utility between these two models. If the default changes, the surrounding consent situation changes; thus, the average utility is expected to show different values. Therefore, we assume the consent rate in the opt-in and opt-out models as $p_{in}$ and $p_{out}$, respectively, with $\;0\leq p_{in}\leq p_{out}\leq 1$ due to the default difference. The difference in consent rates between these two models, *ΔU*, is given by the following equation:

$$\begin{array}{rl} \Delta U & =U_{out}(\;p_{out})-U_{in}(\;p_{in})=\frac{1}{n}\underset{s_{i}=\mathrm{disagree}}{\sum }u_{out}(\;p_{out},q_{i},s_{i})\\ & \;+p_{out}k_{out}-\frac{1}{n}\underset{s_{i}=\mathrm{agree}}{\sum }u_{in}(\;p_{in},q_{i},s_{i})-(1-p_{in})k_{in} \end{array}$$

Here, in the opt-out model, if “disagreeing” is chosen, it satisfies

$$u_{out}(\;p_{out},q_{i},\mathrm{disagree})\geq k_{out}.$$

While in the opt-in model, if “agreeing” is chosen, it satisfies

$$u_{in}(\;p_{in},q_{i},\mathrm{agree})\geq k_{in}.$$

Using these, we get

$$\Delta U\geq k_{out}-\frac{1}{n}\sum_{i=1}^{n}u_{in}(\;p_{in},q_{i},\;\mathrm{agree}).$$

This is positive if the average utility when everyone chooses to “agree” in the opt-in model is below $k_{out}$. Since $\;u_{in}(\;p_{in},q_{i},\;\mathrm{agree})=k_{out}$ until $q_{i}$ satisfies $\;u_{in}(\;p_{in},q_{i},\;\mathrm{agree})<k_{out}$, and considering $u_{in}(\;p_{in},q_{i},\;\mathrm{agree})\geq k_{in}$ if $p_{in}$ is relatively low and there are few subjects with high $q_{i}$, ΔU≥0 is satisfied. Conversely, if $p_{in}$ is high and there are many subjects with high $q_{i}$, ΔU≤0 is satisfied. Assuming the former case, ΔU≥0, indicating that the average utility of the opt-out model is higher.

This mechanism, by which people follow the default choice, has been called the “default effect (24).” If this premise is correct, in the opt-out model, people automatically choose to “agree,” theoretically resulting in a 100% consent rate unless they explicitly indicate “disagreeing.”

However, the opt-out model has a fundamental flaw in that it does not consider the ethical aspects of consent acquisition, namely quality. Typically, informed consent places the highest importance on respecting patient autonomy by ensuring that the patient has the freedom of choice (25). However, this model simplifies the process, which may lead to distrust and doubts from patients. Additionally, medical staff may be concerned about being held responsible for issues such as information leaks or privacy violations due to differences from previous practices (23). Therefore, in societies like Japan, where the opt-in model is already customary, transitioning to the opt-out model is challenging.

Based on the above considerations, Table 1 reexamines the consent models from the perspectives of default settings, efficiency, and quality.

**Table 1:** Relationship between default, efficiency, quality, and consent rate (utility) in each model.

Consent model	Default	Efficiency	Quality	Consent rate (utility)
Opt-in model	Disagree	Low	High	Low
Opt-out model	Agree	High	Low	High
Two-step consent model	Step 1: agree Step 2: disagree	High	High	High to medium

Source: Created by the authors.

### 2.6 Definition of the two-step consent model

As mentioned above, the opt-in and opt-out models face a trade-off between the efficiency and quality of consent acquisition. To overcome these limitations, this study proposes a new two-step consent model as a compromise between these two models (bottom of Table 1). Our model involves preregistering patient information through implied consent via in-hospital notices and obtaining explicit consent from the patient when the medical staff needs to access the patient information. The details of this model are as follows:

$2step_{1}$: first step of consent acquisition.

In the first step, implied consent is obtained in advance through in-hospital notices and public relations to register patient information. The default setting at this stage is set to “agree.” Similar to the opt-out model, medical staff do not directly approach patients to explain and obtain consent, thus reducing consent costs (26).

$2step_{2}$: second step of consent acquisition.

In the second step, explicit consent is obtained from patients during their visits when the medical staff needs to access patient information. The default setting at this stage is set to “disagree.” Notably, at this stage, the medical staff directly obtains consent from the patient using the opt-in model. Therefore, the quality of consent acquisition is ultimately ensured. This preparation is expected to be particularly effective in urgent situations (26).

Based on these assumptions, we define the following variables to formulate this model:

$p_{2step1}$: Consent rate in the first step $\left(0\leq p_{2step1}\leq 1\right)$,

$p_{2step2}$: Consent rate in the second step $\left(0\leq p_{2step2}\leq 1\right)$,

$n_{1}$: Number of subjects in the first step,

$n_{2}$: Number of subjects in the second step $\left(n_{2}=[p_{2step1}n_{1}]\right)$,

$U_{2step1}(p)$: Average utility in the first step when the consent rate is p,

$U_{2step2}(p)$: Average utility in the second step when the consent rate is p*,*

$U_{2step}(p_{2step1},p_{2step2})$: Utility of the two-step consent model.

Using these, we define the following equations:

$$U_{2step1}(p)=\frac{1}{n_{1}}\sum_{i=1}^{n_{1}}\max\{u_{out}(\;p_{2step1},q_{i},s_{i}),\;k_{out}\},$$

$$U_{2step2}(p)=\frac{1}{n_{2}}\sum_{i=1}^{n_{2}}\max\{u_{in}(\;p_{2step2},q_{i},s_{i}),\;k_{in}\}\;$$

Owing to the difference in default settings, the first and second steps are given by $\;u_{out}$ and $u_{in},$ respectively. By multiplying these two equations, the utility of the two-step consent model is defined as follows:

$$U_{2step}(p_{2step1},\;p_{2step2})=U_{2step1}(p_{2step1})\times U_{2step2}(p_{2step2}).$$

For example, if the utility function for consent is set to 1 at each step, and the consent rate in the first and second steps is 90% and 80%, respectively, then the consent rate of the two-step consent model can be easily calculated as

$$U_{2step}=U_{2step1}\times U_{2step2}=0.9\times 0.8=0.72.$$

Thus, as a result of setting a default that obtains consent from patients in two steps, the consent rate may be slightly lower than the opt-out model; however, it will ensure the quality of consent acquisition compared with the opt-in model, thereby resolving the trade-off issue.

### 2.7 Order of average utility among the three consent models

Furthermore, we observe that the following inequality holds for the average utility of these three consent models under general conditions:

$$U_{out}(p_{out})\geq U_{2step}(p_{2step1},\;p_{2step2})\geq U_{in}(p_{in}).$$

In fact, $U_{out}\geq U_{2step}$ always holds as follows:

$$\begin{array}{rl} & U_{out}-U_{2step}=\frac{1}{n_{1}}\sum_{i=1}^{n_{1}}\max\;\{u_{out}(\;p_{out},\;q_{i},\;s_{i}),\;\;k_{out}\}\\ & -\frac{1}{n_{1}}\sum_{i=1}^{n_{1}}\max\;\{u_{out}(\;p_{2step1},\;q_{i},\;s_{i}),\;\;k_{out}\}\\ & \times \frac{1}{n_{2}}\sum_{i=1}^{n_{2}}\max\;\{u_{in}(\;p_{2step2},\;q_{i},\;s_{i}),\;\;k_{in}\}\\ & =\frac{1}{n_{1}}\sum_{i=1}^{n_{1}}\;g(\max\;\{u_{out}(\;p_{out},\;q_{i},\;s_{i}),\;\;k_{out}\}\\ & -\;\max\{u_{out}(\;p_{2step1},\;q_{i},\;s_{i}),\;\;k_{out}\}\\ & \times \frac{1}{n_{2}}\sum_{i=1}^{n_{2}}u_{in}(\;p_{2step2},\;q_{i},\;s_{i})\geq \frac{1}{n_{1}}\sum_{i=1}^{n_{1}}\;\\ & \left(\max\;\{u_{out}(\;p_{2step1},\;q_{i},\;s_{i}),\;\;k_{out}\}\left(1-\frac{1}{n_{2}}\sum_{i=1}^{n_{2}}u_{in}(\;p_{2step2},\;q_{i},\;s_{i})\right)\right)\geq 0. \end{array}$$

Here, the inequality $u_{out}(p_{out},q_{i},s_{i})\geq u_{out}(p_{2step1},q_{i},s_{i})$ (provided that $p_{out}\geq p_{2step1}$) is used. Therefore, the average utility is always higher in the opt-out model. However, when the term inside the parentheses

$$1-\frac{1}{n_{2}}\sum_{i=1}^{n_{2}}u_{in}(p_{2step2},q_{i},s_{i})$$

approaches zero, $U_{2step}$ approaches $U_{out}$. This implies that the subjects who did not agree in the first stage derive utility from agreeing in the second stage. This is expected to be sufficiently satisfied as $q_{i}$ becomes large enough in the second stage. Therefore, $U_{2step}$ provides an average utility close to $U_{out}$. More specific comparisons of average utility are presented in Section 2.8.

Meanwhile, $U_{2step}\geq U_{in}$ does not generally hold, but

$$\begin{array}{rl} U_{2step1}(\;p_{2step1}) & =p_{step1}k_{out}+\frac{1}{n_{1}}\underset{s_{i}=\mathrm{disagree}}{\sum }u_{out}(\;p_{step1},q_{i},\mathrm{disagree})\\ & =p_{step1}k_{out}+\frac{1}{n_{1}}\underset{s_{i}=\mathrm{disagree}}{\sum }u_{out}(\;p_{step1},q_{i},\mathrm{disagree})\\ & \;+(1-p_{step1})k_{out}-\frac{1}{n_{1}}\underset{s_{i}=\mathrm{disagree}}{\sum }k_{out}\\ & =k_{out}+\frac{1}{n_{1}}\underset{s_{i}=\mathrm{disagree}}{\sum }(u_{out}(\;p_{step1},q_{i},\mathrm{disagree})-k_{out})\;\\ & =k_{out}+(1-p_{step1})\frac{1}{\#\{s_{i}=\mathrm{disagree}\}}\\ & \;\times \underset{s_{i}=\mathrm{disagree}}{\sum }(u_{out}(\;p_{step1},q_{i},\mathrm{disagree})-k_{out})\;\\ & =k_{out}+(1-p_{step1})D_{1} \end{array}$$

Thus, $U_{step1}(p_{step1})\;$ is calculated by having $k_{out}$ as the baseline and adding the utility of non-consenting subjects. Here, the term is summarized, as

$$D_{1}=\frac{1}{\#\{s_{i}=\;\mathrm{disagree}\}}\underset{s_{i}=\mathrm{disagree}}{\sum }(u_{out}(p_{step1},q_{i},\;\mathrm{disagree})-k_{out})$$

is the average value of $u_{out}(p_{step1},q_{i},\;\mathrm{disagree})-k_{out}$ for non-consenting subjects, satisfying $0\leq D_{1}\leq 1-k_{out}$. Applying similar transformations to $U_{step2}(p_{step2})$, we obtain

$$U_{2step2}(p_{2step2})=k_{in}+p_{step2}D_{2}.$$

Thus, $U_{step2}(p_{step2})$ has $k_{in}$ as the baseline, with the utility of agreeing subjects added. However,

$$D_{2}=\frac{1}{\#\{s_{i}=\;\mathrm{agree}\}}\underset{s_{i}=agree}{\sum }(u_{in}(p_{step2},q_{i},\;\mathrm{agree})-k_{in})$$

satisfying $0\leq D_{2}\leq 1-k_{in}$. Using these, we obtain

$$U_{2step}=(k_{out}+(1-p_{step1})D_{1})(k_{in}+p_{step2}D_{2}).$$

Applying similar calculations to $U_{in}$, we obtain

$$U_{in}(p_{in})=k_{in}+p_{in}D_{in},$$

$$D_{in}=\frac{1}{\#\{s_{i}=\;\mathrm{agree}\}}\underset{s_{i}=\mathrm{agree}}{\sum }(u_{in}(p_{in},q_{i},\;\mathrm{agree})-k_{in}).$$

For $U_{2step}\geq U_{in}$ to hold, the following must be satisfied:

$$(k_{out}+(1-p_{step1})D_{1})(k_{in}+p_{step2}D_{2})\geq k_{in}+p_{in}D_{in}.$$

Here, since the relationships among $D_{1},D_{2},D_{in}$ vary, this does not always hold. However, if

$$p_{in}D_{in}\leq p_{step2}D_{2}(⋆)$$

is satisfied, $k_{in}$ can be chosen from the interval satisfying

$$\frac{1+p_{step2}D_{2}}{1+p_{in}D_{in}}-(1-p_{step1})D_{1}\leq k_{out}\leq 1-(1-p_{step1})D_{1}$$

regardless of the value of $k_{out}$, ensuring $U_{2step}\geq U_{in}\;$(see Supplementary Material for details). Since $\;D_{2},\;D_{in}$ are almost the same value, (⋆) is sufficient if $p_{in}\leq p_{step2}\;$ is satisfied. This means that the consent rate in the opt-in model is lower than that in the second stage, which is expected to be sufficiently satisfied. However, these are only sufficient conditions, and not satisfying them does not necessarily mean that $U_{2step}\leq U_{in}$.

### 2.8 Numerical experimental examples

Finally, we conducted numerical experiments on these three models; the results are presented in this section. As real-world patient populations have different characteristics, the factors influencing the choice of consent may differ. Therefore, considering patient diversity, we assume representative distributions A-D to ascertain the average utility of each model. For details on the functions and parameters used in these numerical calculations and the shapes of the distributions, see Supplementary Material. Based on the above, Table 2 shows the results of calculating the average utility and average consent rate for each model for five cases, consisting of commonly assumed Cases 1, 2, and 3, and exceptional Cases 4 and 5.

**Table 2:** Numerical calculation examples.

	Opt-in model		Two-step consent model		Opt-out model	
	Utility	Consent rate	Utility	Consent rate	Utility	Consent rate
Case 1	0.533	0.097	0.778	0.929	0.926	0.655
Case 2	0.536	0.102	0.769	0.993	0.903	0.954
Case 3	0.690	0.472	0.758	0.988	0.904	0.942
Case 4	0.532	0.096	0.493	0.649	0.926	0.649
Case 5	0.704	0.498	0.488	0.948	0.904	0.942

Source: Authors’ creation.

Case 1: The distribution of patients in the first and second phases follows distributions A and D, respectively.

Case 2: The distribution of patients in the first and second phases follows distributions B and D, respectively.

Case 3: The distribution of patients in the first and second phases follows distributions C and D, respectively.

Case 4: The distribution of patients in the first and second phases follows distribution A.

Case 5: The distribution of patients in the first and second phases follows distributions C and A, respectively.

The results of the numerical experiments show that the relationship $U_{in}\leq U_{2step}\leq U_{out}$ is established in Cases 1, 2, and 3, with the two-step consent model, in particular, showing a high consent rate. However, in Cases 4 and 5, $U_{2step}\leq U_{in}$, which is a specific uncommon situation wherein the respondents do not feel the need for medical care at the second step.

In all of the above situations, the opt-out model automatically obtains a large number of consents and has the highest utility, since it leads to the selection of “I agree.” Although the opt-out model is superior in terms of its low cost, it includes the portion of “consent” that is considered “consent” without the subject's knowledge. By contrast, the utility and consent rate are low in the opt-in model. It can be seen that this is inefficient because of the cost of consent. Meanwhile, the proposed two-step consent model is cost-effective since the utility and consent rate are comparable to those of the opt-out model, except for Case 4, even though the subject is aware of and gives their consent.

## 3 Results

### 3.1 Online survey

An online survey was conducted from late January to early February 2024, using a panel of 2.2 million monitors managed by one of the largest online survey companies in Japan. To collect a target sample size of 2,000, which aligns with Japan's population distribution, the questionnaire was distributed after prior allocation by gender, age group, and prefecture. Consequently, we obtained a sufficiently randomized sample with a valid collection of 2,000 samples (100%). The questionnaire received prior ethical review from the “Ethical Review Committee for Research Involving Human Subjects” of the Kitami Institute of Technology (October 23, 2018, Approval No.: 1015, July 27, 2022, Approval: KIT-2022-01, Kitami Institute of Technology).

Based on the concept of the contingent valuation method (CVM), we presented the monitors with questions that assumed four situations related to obtaining consent and asked them to respond regarding the acceptability of these situations. The following four situations and symbols were assumed for each model:

$P_{inS}:\;$A scenario assuming administrative staff in the opt-in model,

$P_{inD}:\;$A scenario assuming a physician in the opt-in model,

$P_{out}:\;$A scenario assuming the opt-out model,

$P_{2step}:\;$A scenario assuming the second stage of the two-step consent model.

Here, the response to the opt-in model is divided into two scenarios: administrative staff and physicians, considering that the trust relationship between the medical staff and patients may also influence the response (27). The details of the questions and response items are shown in Supplementary Material. Additionally, a goodness-of-fit test was conducted to confirm whether the differences in opinions for each scenario were statistically significant. The expected ratios were set assuming a normal distribution.

The attribute information of the 2,000 samples was as follows. In terms of gender, 49.5% were males and 50.6% were females. The average age was 50.24 years, with 13.6% in their 20s, 15.8% in their 30s, 20.0% in their 40s, 16.8% in their 50s, 18.7% in their 60s, and 15.3% in their 70s. The residential locations of the monitors are described in Supplementary Material.

### 3.2 Survey results

**Figure 2:**
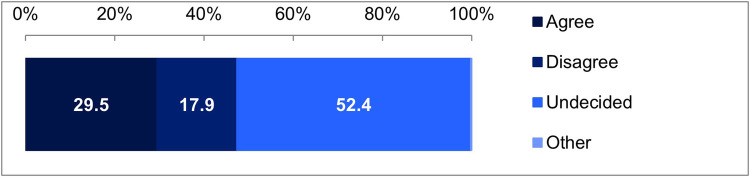
Results for the scenario assuming administrative staff in the Opt-in model. Goodness-of-fit test, *χ*² (1) = 1,831.1536, *p* < 0.001.

The survey results suggested that differences in the distribution of responses in all scenarios were statistically significant. First, we asked about the scenario in which administrative staff handled the opt-in model. The results were as follows: 29.5% of the respondents chose “I think I would sign,” 17.9% chose “I think I would not sign,” 52.4% chose “I don't know if I would sign or not,” and 0.3% chose “Other” (Figure 2). This indicates that the most common response was the ambiguous “I don't know if I would sign or not.”

**Figure 3:**
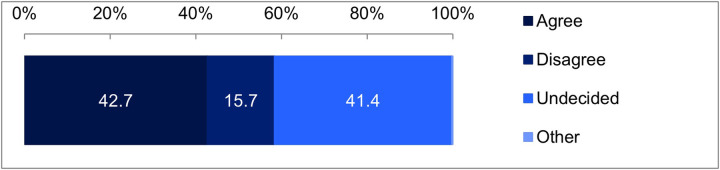
Results for the scenario assuming a physician in the Opt-in model. Goodness-of-fit test, *χ*² (1) = 1,657.8201, *p* < 0.001.

Next, we asked about the scenario in which a physician handled the opt-in model. The results were as follows: 42.7% of the participants chose “I think I would sign,” 15.7% chose “I think I would not sign,” 41.4% chose “I don't know if I would sign or not,” and 0.7% chose “Other” (Figure 3). This indicates that when a physician is involved, the response “I think I would sign” increases by about 10%, while the ambiguous response “I don't know if I would sign or not” decreases by 10%.

**Figure 4:**
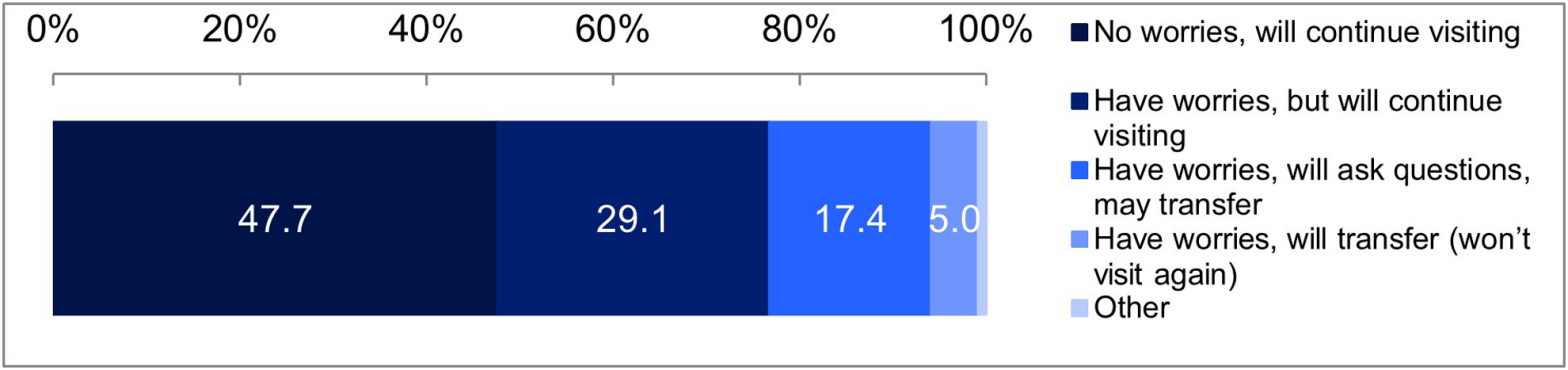
Results for the scenario assuming the Opt-out model. Goodness-of-fit test, *χ*² (1) = 2,324.9391, *p* < 0.001.

Additionally, we asked about the scenario assuming the opt-out model. The results were as follows: 47.7% of the respondents chose “No worries, will continue visiting,” 29.1% chose “Have worries, but will continue visiting,” 17.4% chose “Have worries, will ask questions, may transfer,” 5.0% chose “Have worries, will transfer (won't visit again),” and 0.9% chose “Other” (Figure 4). In this model, unless explicit dissent is expressed, consent is assumed. Therefore, if the 5.0% who answered “Have worries, will transfer (won't visit again)” are considered dissenters, 95% are considered to have consented. However, the number of consenters might decrease depending on the response of the medical staff, as 17.4% answered “Have worries, will ask questions, may transfer.”

**Figure 5:**
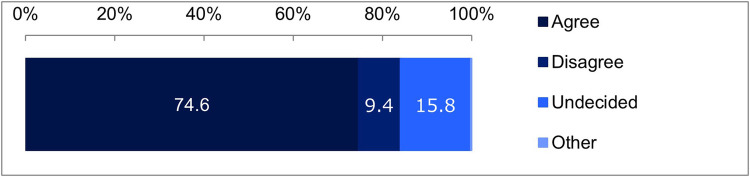
Results for the scenario assuming the second stage of the Two-step consent model. Goodness-of-fit test, *χ*² (2) = 803.0122, *p* < 0.001.

Finally, we asked about the scenario assuming the second stage of the two-step consent model. The results were as follows: 74.6% chose “I think I would sign,” 9.4% chose “I think I would not sign,” 15.8% chose “I don't know if I would sign or not,” and 0.3% chose “Other” (Figure 5). This indicates that in the two-step consent model, the proportion of the ambiguous response “I don't know if I would sign or not” was lower than in the opt-out model, while the proportion of the “I think I would sign” response was higher than in the opt-in model.

Among the four scenarios, it is clear that the opt-out model has the highest likelihood of obtaining signatures. However, approximately one-third of respondents answered, “I have worries or concerns, but I will continue visiting,” indicating that it is not unconditionally acceptable. By contrast, in the two-step consent model, about three-quarters of respondents answered, “I think I would sign,” suggesting that it is generally well-received.

### 3.3 Estimated consent rates

**Figure 6:**
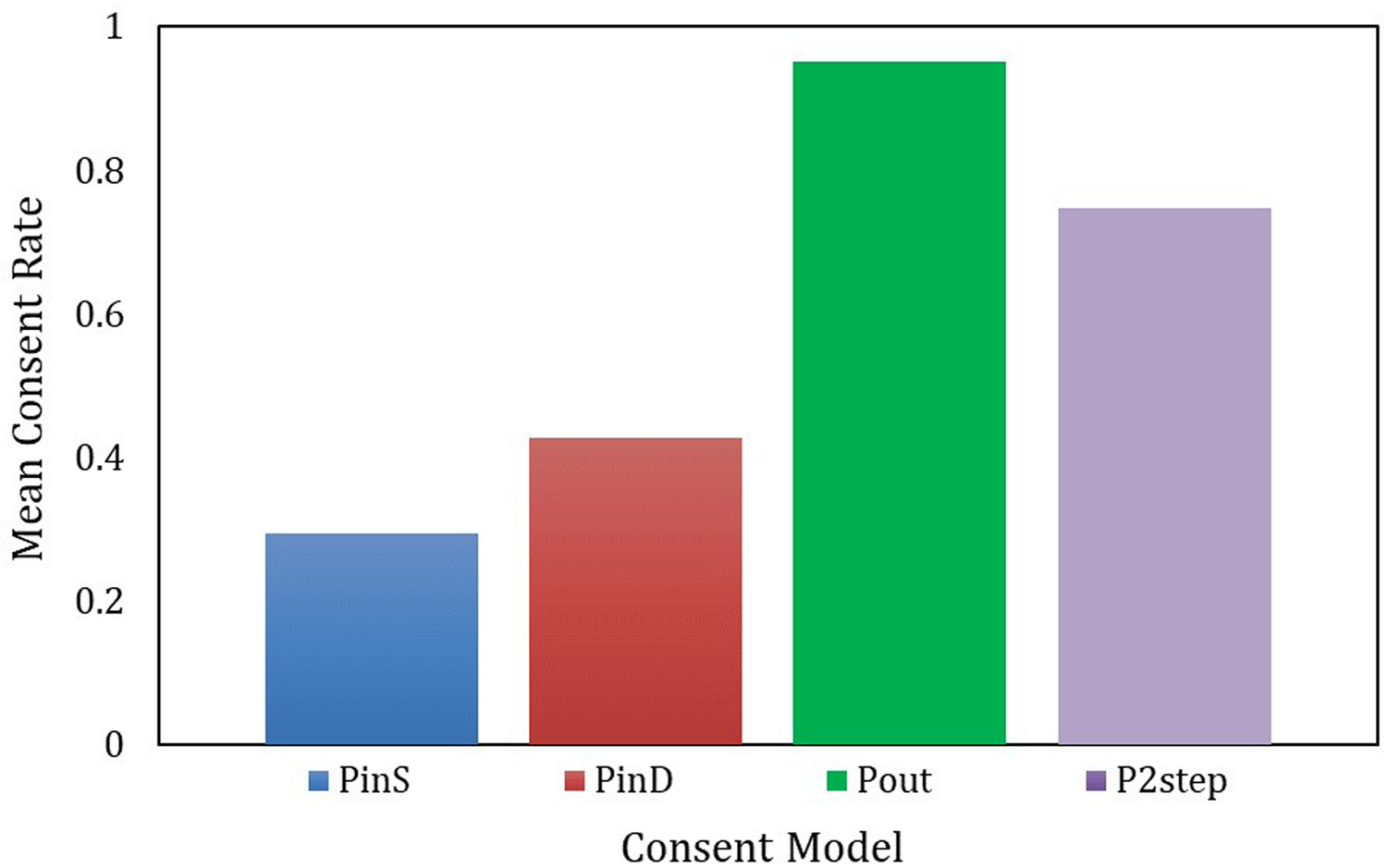
Mean value graph of consent rates by model. One-way ANOVA, F (3,7996) = 1,033.020, *p* < 0.000.

Based on these results, we estimated the consent rates for each scenario. Figure 6 shows the mean value graph, and Table 3 shows the descriptive statistics. We converted the responses into dummy variables to estimate the consent rates: for $P_{inS}$,$P_{inD}$, and $P_{2step}$, “I think I would sign” = 1, and “Other” = 0; for $P_{out}$, “I have worries or concerns, so I will consider transferring (never visit again)” = 0, and “Other” = 1. Note that the first stage of $P_{2step}$ uses the values from $P_{out}$.

**Table 3:** Descriptive statistics.

Consent model	Mean	SD	Min	Max	Median	CI (min-max)
*P_inS_*	0.295	0.456	0	1	0	0.275–0.315
*P_inD_*	0.427	0.495	0	1	0	0.405–0.448
*P_out_*	0.950	0.218	0	1	1	0.940–0.960
*P_2step_*	0.685	0.435	0	1	1	0.666–0.704

Source: Created by the authors.

The analysis revealed that the relationship $U_{out}\geq U_{2step}\geq U_{in}$ derived from the models in Chapter 2 holds true (Figure 6). To determine whether the differences in the means of these four values are statistically significant, a one-way analysis of variance (ANOVA) was conducted, with the results highlighting a significant difference [F (3, 7996) = 1,033.020, *p* < 0.000]. Descriptive statistics are shown in Table 3 for further details.

First, $P_{inS}$ had a low mean of 0.295 and a relatively large standard deviation of 0.456, indicating considerable variability in the consent rate. The median was 0, suggesting that more than half of the respondents tended not to consent. Next, $P_{inD}$ had a higher mean of 0.427 and a slightly larger standard deviation of 0.495, compared to $P_{inS}$, indicating that despite the considerable variability in the consent rate, there was a higher tendency to consent. However, the median was 0, indicating that even when a physician was involved, more than half of the respondents did not consent.

Furthermore, regarding $P_{out}$, the average was 0.950, the highest, and the standard deviation was 0.218, the smallest, indicating a consistently high agreement rate among the subjects. Additionally, the effectiveness ratios with the opt-in model, $P_{out}$/$P_{inS}$ = 3.220 and $p_{out}$/$P_{inD}$ = 2.225 suggest that $P_{out}$ is the most efficient. Finally, $P_{2step}$ had an average of 0.685 and a standard deviation of 0.435, indicating a lower average and greater variability compared with $P_{2}$. However, the effectiveness ratios with the opt-in model, $P_{2step}$/$P_{inS}$ = 2.322 and $P_{2step}$/$P_{inD}$ = 1.604, were both above 1, confirming its efficiency.

In summary, the most efficient model is $P_{out}$, followed by the $P_{2step}$ model. Conversely, $P_{inS}$ and $P_{inD}$ had low agreement rates, suggesting that even if the person obtaining consent was a physician instead of administrative staff, there is a limit to the improvement in agreement rates.

## 4 Discussion

In this study, we raised the question: can the stagnant consent rate be overcome by setting defaults that balance both the efficiency and quality of consent acquisition? To answer this question, we formulated a new two-step consent model using utility theory and evaluated its acceptability along with that of the traditional model. The results show that the opt-in model has limitations in improving the consent rate, even if the physician responds, and that the opt-out model may cause the highest level of distrust on the part of the patient. Meanwhile, the proposed two-step consent model showed an intermediate consent rate between the opt-in and opt-out models, confirming that it can balance both efficiency and quality in obtaining consent. In this section, we will discuss the limitations of the conventional model and how the two-step consent model may overcome these limitations.

### 4.1 Advantages and limitations of the Opt-in model

First, we examined the Opt-in model, which sets the default consent option to “disagree.” While the opt-in model is expected to improve consent rates when handled by physicians rather than administrative staff, it has limitations as a means of improving consent rates due to its impact on clinical efficiency. Indeed, the consent rate was slightly over 10% higher when handled by physicians (42.7%) than when handled by administrative staff (29.5%). Therefore, if physicians attempt to persuade patients directly from a medical standpoint, the low consent rates can be improved while ensuring the quality of consent acquisition.

However, owing to the nature of this model, the cost of obtaining consent becomes significant, potentially negatively affecting the physicians' primary clinical duties. In fact, Japan has a higher number of outpatient visits and a higher frequency of visits than other countries, making it difficult for physicians to spend sufficient time explaining medical situations and obtaining consent from patients (28). Therefore, it is not reasonable for physicians to spend a significant amount of their time to obtain consent from patients. Moreover, this could contradict the recent work style reforms for physicians, which the Japanese government is focusing on at present. Further, while increasing the number of administrative staff or enhancing public relations to promote consent acquisition are possible options, they require budget adjustments. Additionally, this model has challenges from a user experience perspective (29). If patients have no experience using HIE, even if medical staff attempt to obtain consent, they may not be able to comprehend its benefits, thereby making it less persuasive.

### 4.2 Advantages and limitations of the opt-out model

Next, we examined the opt-out model, where the default consent option is set to “agree.” In Japan's medical settings, where the opt-in method is path-dependent, fundamentally changing the default to an opt-out model may be difficult for both patients and medical institutions to accept. Indeed, the consent rate for the opt-out model demonstrated the highest level at 95.0%, which was 2.2–3.2 times higher than that of the opt-in model. According to a survey in England, 94% of respondents were satisfied with the secondary use of medical data obtained through the opt-out method, which aligns with the results of this study (30). Therefore, this model may be considered the most efficient in terms of effectiveness. However, looking at the detailed survey results in Figure 3, 29.1% of respondents continued their visits despite having concerns or worries, and 17.4% considered changing hospitals depending on the answers to their questions. Combined, nearly half of the respondents had concerns, which cannot be ignored. This suggests that respondents might change their consent status depending on the situation. The reasons for the distrust and doubts among respondents included serious issues where the qualitative aspects of consent acquisition were not guaranteed.

Recently, the Japanese government has been promoting implied consent to improve the consent rate for HIEs (31). However, the transition to the opt-out model in medical settings has not progressed, possibly due to concerns about registering personal information in HIEs amidst ongoing incidents of system downtimes and information leaks caused by cyberattacks (32, 33). Additionally, some patients only use medical institutions to consult their primary care physician. If there is no need to use multiple medical institutions, the actual use may not increase even if the opt-out model superficially raises the consent rate. Moreover, in Japan's medical settings, obtaining consent through the opt-in method has already become the norm, making it difficult to overturn such practices fundamentally.

Therefore, while the opt-out model is superior in terms of the efficiency of consent acquisition, it has limitations due to the potential for superficial use caused by qualitative issues.

### 4.3 Acceptability of the two-step consent model

Furthermore, we evaluated the Two-Step Consent model. In this approach, the default for initial consent is set to “agree,” thereby obtaining implied consent. At a later stage, when a physician requires access to patient information, explicit consent is obtained by setting the default to “disagree.” This model effectively leverages the advantages of both the Opt-In and Opt-Out models. Based on the results, the proposed two-step consent model has the potential to improve consent rates through default settings that balance both the efficiency and quality of consent acquisition. Indeed, while the consent rate for this model is 68.5%, which is 26.5% lower than the opt-out model, only 9.4% of respondents answered that they “disagree.” As previously mentioned, cyberattacks and data breaches targeting hospitals have been frequent in Japan (32, 33). Strengthening measures to address these vulnerabilities may improve consent rates to levels comparable to those achieved by the opt-out model. Thus, this model is considered cost-effective as it can overcome the limitations of previous methods by leveraging the benefits of both opt-in and opt-out methods.

Furthermore, this model offers significant benefits for medical institutions. Although the HIE is a rational system that contributes to society as a whole, in reality, few medical institutions are connected to HIEs. One reason is that when hospitals are in a competitive relationship, the medical information and information systems accumulated by hospitals become important strategic management resources (34), which are not necessarily in line with economic rationality. In other words, hospitals may be concerned about the risk of losing patients to other hospitals or having valuable information extracted. However, in this model, patient information can only be viewed when the patient visits the hospital and the physician examines the patient. In other cases, access to other patient information is restricted, alleviating hospital concerns about opportunistic behavior, such as unauthorized viewing or extraction of information. Therefore, it is essential to implement measures that align with the economic rationality of hospitals in advance to gain cooperation from hospitals. In this regard, the initiatives of Inland Empire Health Plan (IEHP), the largest Medicaid plan in the United States, are instructive (35). This organization has introduced financial incentives for medical institutions that share patient data across institutional boundaries, improving utilization rates by compensating for management.

In conclusion, the two-step consent model can overcome the shortcomings of traditional models through the reconfiguration of default settings, making it a valuable policy option for future implementation. However, ensuring the robust adoption of this model necessitates a focus on patient education and comprehension.

### 4.4 Limitations and future research directions

Finally, we summarize the limitations of this study. First, although we proposed and verified the acceptability of the two-step consent model, we did not delve into the specific characteristics of the patients. Since the preferences and medical needs of the subjects vary (36), identifying utilities according to attributes and needs by future research may allow for more effective strategies. For instance, even with the two-step consent model, 15% of respondents showed ambiguous reactions, indicating that they were unsure whether they would consent. Similar negative reactions have been reported in surveys from other fields (37). Therefore, while it may not be realistic to achieve a 100% consent rate, examining the details of such ambiguous reactions and refusals in the future could help determine a reasonable level of consent rates.

Next, this study did not discuss information security or privacy protection measures aimed at improving consent rates. Further, it did not explore the effect of budgetary constraints on individual responses at the medical institution level. Therefore, future research should consider support measures at the policy level. Furthermore, this model assumed a constant number of subjects; however, the actual scale of information networks varies, and the number of subjects differs. Since the number of subjects affects the utility and consent rates of patients, future analyses should focus on the number of subjects and the scale of information networks.

Addressing the stagnation of HIE requires more than just improving consent rates. It is imperative to ensure operational cost-effectiveness, EHR standardization, interoperability, privacy, availability, and reliability to build trust among hospitals and encourage information sharing. These policy issues warrant further discussion.

Nevertheless, this study not only establishes the relationship between consent mechanisms and defaults using utility theory but also proposes a new consent model that overcomes the shortcomings of previous methods of obtaining consent. This model quantitatively demonstrates that it can guarantee both efficiency and quality in obtaining consent. Previous studies viewed the opt-in and opt-out approaches as mutually exclusive, which limited their ability to balance the efficiency and quality of consent acquisition. Conversely, this study introduces a new model that resolves these shortcomings with its novel results, thus making significant contributions to the extant literature as well as healthcare policymaking.

## 5 Conclusion

This study aimed to overcome the issue of low consent rates in HIEs by mathematically formulating a consent mechanism and proposing a new two-step consent model. We verified its acceptability through an online survey. The results showed that the acceptability of this model reached about 70%, indicating its potential for improving consent rates while balancing the efficiency and quality of consent acquisition compared with traditional models. On the one hand, the traditional opt-in model, while maximizing patient autonomy by having medical staff directly explain medical situations and obtain explicit consent, faced issues of increased consent burden and decreased clinical efficiency, with acceptability falling below 50%. On the other hand, the opt-out model, despite being the most efficient approach, was found to be fundamentally flawed, as approximately half of the respondents had some concerns. The proposed two-step consent model initially obtains implicit consent and then explicit consent when the medical staff needs to access patient information, thus efficiently obtaining consent while ensuring patient autonomy. By leveraging the default effect in this way, it can become a cost-effective policy option. Moving forward, rather than viewing opt-in and opt-out as opposing approaches, we hope that the introduction of new methods that leverage the advantages of both methods can help overcome stagnation issues in HIEs. Educating patients on the significance of HIE and the two-step consent model is critical to fostering understanding and acceptance.

## Data Availability

The original contributions presented in the study are included in the article/Supplementary Material, further inquiries can be directed to the corresponding author.

## Footnotes

^1^“In reality, the set of options also includes choices such as ‘undecided’ and ‘unaware.’ For example, if the default is set to ‘agree/disagree,’ selecting ‘undecided’ or ‘unaware’ would be evaluated for both ‘agree’ and ‘disagree,’ and the higher utility would be chosen.”

## References

[1] Sarkar IN. Transforming health data to actionable information: recent progress and future opportunities in health information exchange. Yearbook Med Inform. (2022) 31:203–14. 10.1055/s-0042-1742519 PMC9719753 36463879

[2] Menachemi N Rahurkar S Harle CA Vest JR. The benefits of health information exchange: an updated systematic review. J Am Med Inform Assoc. (2018) 25:1259–65. 10.1093/jamia/ocy035 29718258 PMC7646861

[3] Payne TH Lovis C Gutteridge C Pagliari C Natarajan S Yong C Status of health information exchange: a comparison of six countries. J Glob Health. (2019) 9:0204279. 10.7189/jogh.09.020427 31673351 PMC6815656

[4] Ito A Okumura T. Factors for stagnation in the regional healthcare networks project: analysis of initial investment and management model. Kaikei-Kensa Kenkyu. (2021) 64:63–84.

[5] Shimbun NK. Sharing of medical data becomes hollow. (2019). Available online at: https://www.nikkei.com/article/DGXMZO42441870U9A310C1SHA000/ (Accessed March 20, 2024).

[6] Esmaeilzadeh P Sambasivan M. Patients’ support for health information exchange: a literature review and classification of key factors. BMC Med Inform Decis Mak. (2017) 17:33. 10.1186/s12911-017-0436-2 28376785 PMC5379518

[7] Ministry of Health, Labour and Welfare. Examples of consent acquisition methods in regional medical information collaboration networks (2020).

[8] Ai W. Overview of National Regional Medical Information Collaboration Network Using ICT (2022 Edition), Nichii soken Working Paper, No. 475 (2022). Available online at: https://www.jmari.med.or.jp/result/working/post-3866/ (Accessed March 20, 2024).

[9] Hill D Walker J Hale J. Privacy Considerations for Health Information Exchanges, Medical Data Privacy Handbook. Cham: Springer (2015). p. 289–311.

[10] Suzumoto J Mori Y Kuroda T. Health information exchange use by healthcare workers in Japan: retrospective cohort study. JMIR Med Inform. (2024) 12(1):e56263. 10.2196/preprints.56263 39382566 PMC11481819

[11] Chimonas S Lipitz-Snyderman A Matsoukas K Kuperman G. Electronic consent in clinical care: an international scoping review. BMJ Health Care Inform. (2023) 30:e100726. 10.1136/bmjhci-2022-100726 37423643 PMC10335420

[12] Esmaeilzadeh P Mirzaei T. Comparison of consumers’ perspectives on different health information exchange (HIE) mechanisms: an experimental study. Int J Med Inform. (2018) 119:1–7. 10.1016/j.ijmedinf.2018.08.007 30342677

[13] Samuelson W Zeckhauser R. Status quo bias in decision making. J Risk Uncertain. (1988) 1(1):7–59. 10.1007/BF00055564

[14] Tversky A Kahneman D. Loss aversion in riskless choice: a reference-dependent model. Q J Econ. (1991) 106(4):1039–61. 10.2307/2937956

[15] Jachimowicz JM Duncan S Weber EU Johnson EJ. When and why defaults influence decisions: a meta-analysis of default effects. Behav Public Policy. (2019) 3:159–86. 10.1017/bpp.2018.43

[16] OECD. Behavioral Public Policy: New Policy Design Utilizing Insights from Behavioral Economics. Tokyo: Akashi Shoten (2016). p. 61–87.

[17] Dhingra N Gorn Z Kener A Dana J. The default pull: an experimental demonstration of subtle default effects on preferences. Judg Decis Mak. (2012) 7:69–76. 10.1017/S1930297500001844

[18] Cabral LMB. Introduction to Industrial Organization. 2nd ed. Cambridge, MA: The MIT Press (2017).

[19] Ito A Nakamura F. Alliance of medical malls: conditions for the establishment of network practice. J Jpn Manag. (2021) 5:1–16.

[20] Ministry of Health, Labour and Welfare. Current status of regional medical information collaboration networks (2020). Available online at: https://www.mhlw.go.jp/content/10800000/000683765.pdf (Accessed March 20, 2024).

[21] Yokohama City Government. *Patient consent and information related to mutual collaboration, corresponding to Chapter 3 of the guidelines*, *Yokohama city ICT utilization regional medical collaboration network guidelines* (2018). Available online at: https://www.city.yokohama.lg.jp/kurashi/kenkoiryo/iryo/seisaku/ICT/guideline.files/0011_20180927.pdf (Accessed March 20, 2024).

[22] de Man Y Wieland-Jorna Y Torensma B de Wit K Francke AL Oosterveld-Vlug MG Opt-In and opt-out consent procedures for the reuse of routinely recorded health data in scientific research and their consequences for consent rate and consent bias: systematic review. J Med Internet Res. (2023) 25:e42131. 10.2196/42131 36853745 PMC10015347

[23] Apathy NC Holmgren AJ. Opt-in consent policies: potential barriers to hospital health information exchange. Am J Manag Care. (2020) 26:e14–20. 10.37765/ajmc.2020.42148 31951362 PMC7262872

[24] Johnson EJ Goldstein D. Medicine – do defaults save lives? Science. (2003) 302:1338–9. 10.1126/science.1091721 14631022

[25] Hall DE Prochazka AV Fink AS. Informed consent for clinical treatment. CMAJ. (2012) 184:533–40. 10.1503/cmaj.112120 22392947 PMC3307558

[26] Medford-Davis LN Chang L Rhodes KV. Health information exchange: what do patients want? Health Inform J. (2017) 23:268–78. 10.1177/1460458216647190 27245671

[27] Kim KK Sankar P Wilson MD Haynes SC. Factors affecting willingness to share electronic health data among California consumers. BMC Med Ethics. (2017) 18:25. 10.1186/s12910-017-0185-x 28376801 PMC5381052

[28] OECD. Consultations with Doctors, Health at a Glance: Asia/Pacific 2022 Measuring Progress Towards Universal Health Coverage. Paris: OECD (2022). p. 106–7.

[29] Bhaskaran V. Designing for trust: the crucial role in digital user experience. J User Experience. (2024) 19:53–9.

[30] Atkin C Crosby B Dunn K Price G Marston E Crawford C Perceptions of anonymised data use and awareness of the NHS data opt-out amongst patients, carers and healthcare staff. Res Involvement Engagement. (2021) 7:40. 10.1186/s40900-021-00281-2 PMC8201435 34127076

[31] Ministry of Health, Labour and Welfare. Mechanisms for obtaining consent (2023). Available online at: https://www.mhlw.go.jp/content/10808000/001045573.pdf (Accessed March 20, 2024).

[32] Hata M. Japanese hospitals increasingly at risk of cyberattacks. The Japan News (2021). Available online at: https://japannews.yomiuri.co.jp/society/crime-courts/20210926-33177/ (Accessed July 20, 2024).

[33] Zhu P Shen J Xu M. Patients’ willingness to share information in online patient communities: questionnaire study. J Med Internet Res. (2020) 22:1–13. 10.2196/16546 PMC7160706 32234698

[34] Kuo KM Liu CF Talley PC Pan SY. Strategic improvement for quality and satisfaction of hospital information systems. J Healthc Eng. (2018) 2018:1–14. 10.1155/2018/3689618 PMC6157169 30298099

[35] Juhn E Galvez E. Incentivizing data sharing among health plans, hospitals, and providers to improve quality. Am J Manag Care. (2022) 28:e426–7. 10.37765/ajmc.2022.89277 36525661

[36] Tong V Krass I Robson S Aslani P. Opt-in or opt-out health-care communication? A cross-sectional study. Health Expect. (2021) 24:731–43. 10.1111/hex.13230 33761176 PMC8235885

[37] Lutomski JE Manders P. From opt-out to opt-in consent for secondary use of medical data and residual biomaterial: an evaluation using the RE-AIM framework. PLoS One. (2024) 19:e0299430. 10.1371/journal.pone.0299430 38547214 PMC10977758

