# Supplementary material for: Consent mechanisms and default effects in health information exchange in Japan
Source: Front Digit Health. 2025 Feb 24;7:1498072. doi: 10.3389/fdgth.2025.1498072 (PMC11908376; doi:10.3389/fdgth.2025.1498072)
Supplement: Supplementary file 1 [file Datasheet1.pdf]

Supplementary Material 1

The functions and parameters used in the numerical calculations, as well as distributions A to D, are listed here.

Utility function : $A = 20, b = 1.0, c = 0.6$

$$u_{in}(p, q, \text{agree}) = \frac{1}{1 + e^{-A(p+q-b)}}, \quad u_{in}(p, q, \text{disagree}) = k_{in} = 0.5$$

$$u_{out}(p, q, \text{disagree}) = \frac{1}{1 + e^{A(p+q-c)}}, \quad u_{out}(p, q, \text{agree}) = k_{out} = 0.9$$

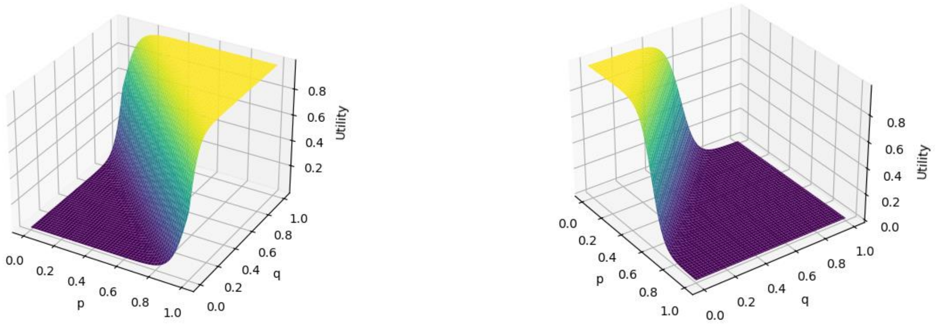

Figure1 （Left） $u_{in}(p, q, \text{agree})$, （Right） $u_{out}(p, q, \text{disagree})$

Distribution A：(0.2, 0.15, 0.15, 0.1, 0.1, 0.1, 0.1, 0.05, 0.03, 0.02)

Distribution B：(0.02, 0.03, 0.05, 0.1, 0.3, 0.3, 0.1, 0.05, 0.03, 0.02)

Distribution C：(0.02, 0.03, 0.05, 0.1, 0.1, 0.1, 0.1, 0.15, 0.15, 0.2)

Distribution D：(0.0, 0.0, 0.0, 0.0, 0.05, 0.05, 0.1, 0.2, 0.3, 0.3)

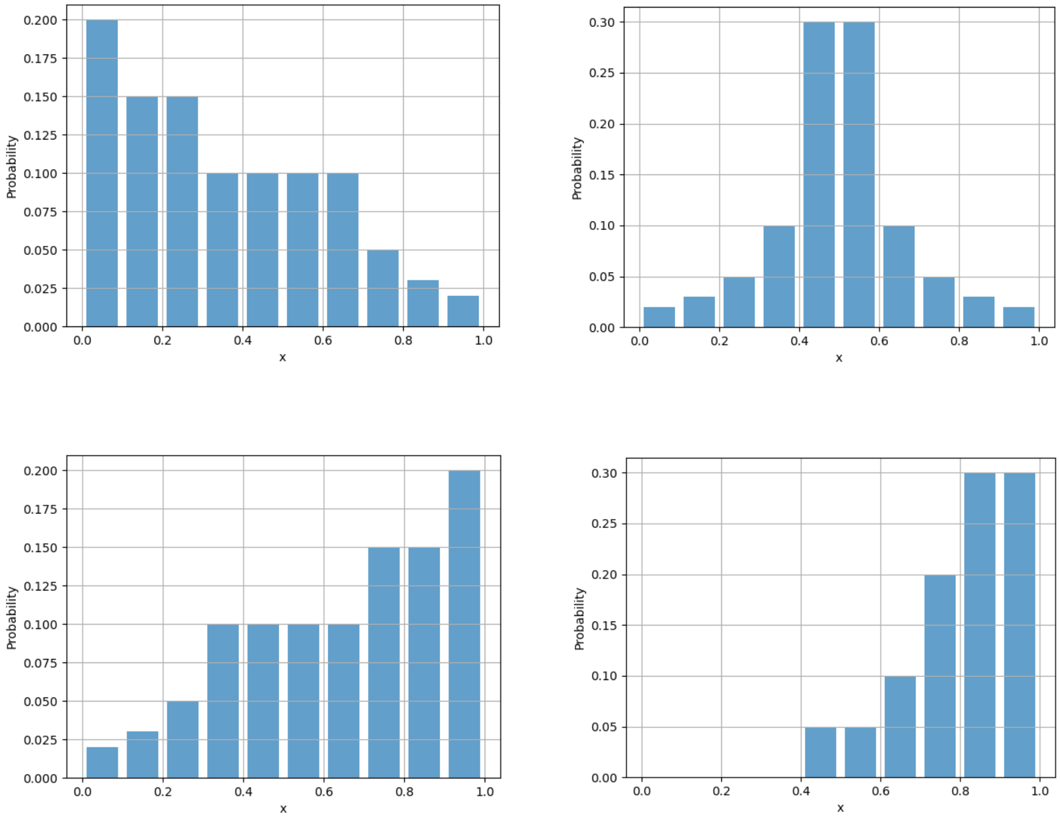

Figure2 (Top Left) Distribution A, (Top Right) Distribution B, (Bottom Left) Distribution C, (Bottom Right) Distribution D

Supplementary Material 2

Lemma. Let $a, b, c, s, t, u \in [0,1]$. There exist $x, y \in [0,1]$ such that

$$(y + as)(x + bt) \geq x + cu.$$

Proof. The inequality can be formed as

$$y \geq \frac{x + cu}{x + bt} - as = 1 - as - \frac{cu - bt}{x + bt}$$

This is a hyperbolic function having asymptote $y = 1 - as$ (< 1). Then, for any $x \in [0,1]$, there exists y such that the inequality holds if

$$\frac{cu - bt}{x + bt} \geq 0,$$

that is, $cu \geq bt$. Note that the notations correspond as $y = k_{out}$, $x = k_{in}$, $a = 1 - p_{step1}$, $b = p_{step2}$, $c = p_{in}$, $s = D_1$, $t = D_2$, $u = D_{in}$.

Supplementary Material 3

Table1 Hypothetical Survey Items

	Person in Charge	Hypothetical Question Content
P_{inS}	Administrative Staff	After you visit the hospital and receive a consultation, while waiting for the payment, an administrative staff member asks, “Do you have a moment?” When you respond, they explain, “We can store your medical information in a network for future use when you visit other medical institutions, are transported by emergency services, or if your medical records are lost in a disaster.” If you are asked to participate in the network in such a situation, would you agree and sign the consent form? Note that the explanation, Q&A, and signing of the consent form will take about 5 to 15 minutes in total.<br>1. I think I would sign<br>2. I think I would not sign<br>3. I don’t know if I would sign or not<br>4. Other
P_{inD}	Doctor	During a regular hospital visit and consultation, the doctor explains, “We can store your medical information in a network for future use when you visit other medical institutions, are transported by emergency services, or if your medical records are lost in a disaster.” If you are asked to participate in the network in such a situation, would you agree and sign the consent form?<br>1. I think I would sign<br>2. I think I would not sign<br>3. I don’t know if I would sign or not<br>4. Other
P_{out}	—	After confirming the following posted content, please select the action that is closest to what you would take.<br>1. I have no particular concerns or complaints, so I will continue visiting the hospital<br>2. I have concerns or complaints, but I will continue visiting the hospital

<table>
<tr><td></td><td></td><td>
3. I have concerns or complaints, so I will ask questions and consider transferring hospitals depending on the answers

4. I have concerns or complaints, so I will transfer hospitals (I will never visit again)

5. Other

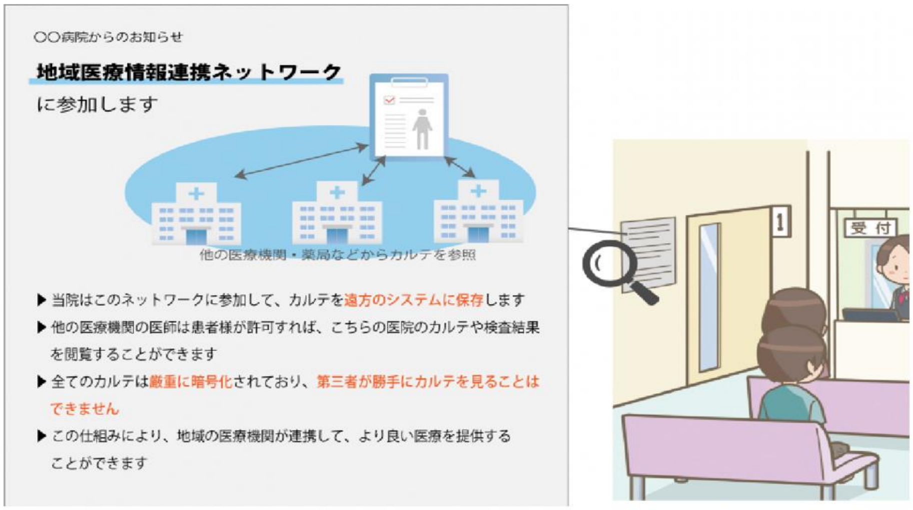

Note: The actual survey questions are presented on a Japanese screen.
</td></tr>
<tr><td>P_{2step}</td><td>Doctor</td><td>
You suddenly experience severe back pain at home and are transported to the hospital by emergency services. The attending doctor in the emergency room asks, “May I view the medical records from the medical institution you are currently visiting for the purpose of diagnosis?” If you were in such a situation, do you think you would agree to let the doctor view your medical records?

1. I think I would sign

2. I think I would not sign

3. I don’t know if I would sign or not

4. Other
</td></tr>
</table>

Source: Created by the authors.

Table2 Sex

	n	%
Total	2000	100.0
Male	989	49.5
Female	1011	50.6

Source: Created by the authors.

Table3 Age

	Values
Total	2000
Mean	50.24
Min	20.00
Max	79.00

Source: Created by the authors.

Table4 Age Group

	n	%
Total	2000	100.0
20s	271	13.6
30s	316	15.8
40s	399	20.0
50s	335	16.8

60s	374	18.7
70s	305	15.3

Source: Created by the authors.

Table5　Sample Size by Prefecture

	n	%
Total	2000	100.0
Hokkaido	84	4.2
Aomori	19	1.0
Iwate	19	1.0
Miyagi	37	1.9
Akita	15	0.8
Yamagata	15	0.8
Fukushima	30	1.5
Ibaraki	47	2.4
Tochigi	31	1.6
Gunma	31	1.6
Saitama	120	6.0
Chiba	100	5.0
Tokyo	228	11.4
Kanagawa	147	7.4

Niigata	36	1.8
Toyama	17	0.9
Ishikawa	17	0.9
Fukui	12	0.6
Yamanashi	12	0.6
Nagano	31	1.6
Gifu	31	1.6
Shizuoka	58	2.9
Aichi	119	6.0
Mie	28	1.4
Shiga	22	1.1
Kyoto	41	2.1
Osaka	142	7.1
Hyogo	86	4.3
Nara	21	1.1
Wakayama	13	0.7
Tottori	11	0.6
Shimane	12	0.6
Okayama	29	1.5
Hiroshima	42	2.1

Yamaguchi	21	1.1
Tokushima	12	0.6
Kagawa	14	0.7
Ehime	22	1.1
Kochi	12	0.6
Fukuoka	80	4.0
Saga	12	0.6
Nagasaki	20	1.0
Kumamoto	26	1.3
Oita	18	0.9
Miyazaki	14	0.7
Kagoshima	24	1.2
Okinawa	22	1.1
Overseas	0	0.0

Source: Created by the authors.
